# Cofactor Tail Length Modulates Catalysis of Bacterial F_420_-Dependent Oxidoreductases

**DOI:** 10.3389/fmicb.2017.01902

**Published:** 2017-09-27

**Authors:** Blair Ney, Carlo R. Carere, Richard Sparling, Thanavit Jirapanjawat, Matthew B. Stott, Colin J. Jackson, John G. Oakeshott, Andrew C. Warden, Chris Greening

**Affiliations:** ^1^School of Biological Sciences, Monash University, Clayton, VIC, Australia; ^2^Land and Water Flagship, The Commonwealth Scientific and Industrial Research Organisation, Acton, ACT, Australia; ^3^GNS Science, Wairakei Research Centre, Lower Hutt, New Zealand; ^4^Department of Microbiology, University of Manitoba, Winnipeg, MB, Canada; ^5^Research School of Chemistry, Australian National University, Acton, ACT, Australia

**Keywords:** F_420_, redox, biocatalysis, biodegradation, mycobacterium, actinobacteria, cofactor

## Abstract

F_420_ is a microbial cofactor that mediates a wide range of physiologically important and industrially relevant redox reactions, including in methanogenesis and tetracycline biosynthesis. This deazaflavin comprises a redox-active isoalloxazine headgroup conjugated to a lactyloligoglutamyl tail. Here we studied the catalytic significance of the oligoglutamate chain, which differs in length between bacteria and archaea. We purified short-chain F_420_ (two glutamates) from a methanogen isolate and long-chain F_420_ (five to eight glutamates) from a recombinant mycobacterium, confirming their different chain lengths by HPLC and LC/MS analysis. F_420_ purified from both sources was catalytically compatible with purified enzymes from the three major bacterial families of F_420_-dependent oxidoreductases. However, long-chain F_420_ bound to these enzymes with a six- to ten-fold higher affinity than short-chain F_420_. The cofactor side chain also significantly modulated the kinetics of the enzymes, with long-chain F_420_ increasing the substrate affinity (lower *K*_m_) but reducing the turnover rate (lower *k*_cat_) of the enzymes. Molecular dynamics simulations and comparative structural analysis suggest that the oligoglutamate chain of F_420_ makes dynamic electrostatic interactions with conserved surface residues of the oxidoreductases while the headgroup binds the catalytic site. In conjunction with the kinetic data, this suggests that electrostatic interactions made by the oligoglutamate tail result in higher-affinity, lower-turnover catalysis. Physiologically, we propose that bacteria have selected for long-chain F_420_ to better control cellular redox reactions despite tradeoffs in catalytic rate. Conversely, this suggests that industrial use of shorter-length F_420_ will greatly increase the rates of bioremediation and biocatalysis processes relying on purified F_420_-dependent oxidoreductases.

## Introduction

Diverse enzymes employ flavins and similar cofactors to mediate biological redox reactions (Leys and Scrutton, 2016). In addition to using the universal flavin cofactors FAD and FMN, some bacteria and archaea employ the deazaflavin cofactor F_420_ (Ney et al., 2017). In its enzyme-unbound state, this redox cofactor has unique redox properties compared to free FMN and FAD, namely a lower standard redox potential (-340 mV) and exclusive two-electron reactivity (Walsh, 1986; Greening et al., 2016). Due to these properties, F_420_ can mediate a wide range of otherwise challenging redox transformations, including the one-carbon reactions of methanogenesis (Thauer et al., 2008; Greening et al., 2016). In mycobacteria and streptomycetes, the cofactor has been shown to be important for central metabolism (Bashiri et al., 2008; Ahmed et al., 2015), secondary metabolite biosynthesis (Ikeno et al., 2006; Wang et al., 2013), cell wall production (Purwantini and Mukhopadhyay, 2013; Purwantini et al., 2016), and biodegradation pathways (Taylor et al., 2010; Jirapanjawat et al., 2016). Beyond its physiological importance, F_420_ has received recent attention for its potential industrial applications. Notably, actinobacterial F_420_H_2_-dependent reductases catalyze the penultimate step of tetracycline antibiotic biosynthesis (Wang et al., 2013), the reductive activation of the clinically approved antituberculosis prodrug delamanid (Cellitti et al., 2012), and the biodegradation of environmental contaminants such as nitroaromatic explosives (Ebert et al., 1999) and arylmethane dyes (Jirapanjawat et al., 2016). F_420_ has also been identified as a promising next-generation cofactor to mediate *in vitro* and *in vivo* biocatalytic cascades (Taylor et al., 2013; Greening et al., 2017).

**FIGURE 1:**
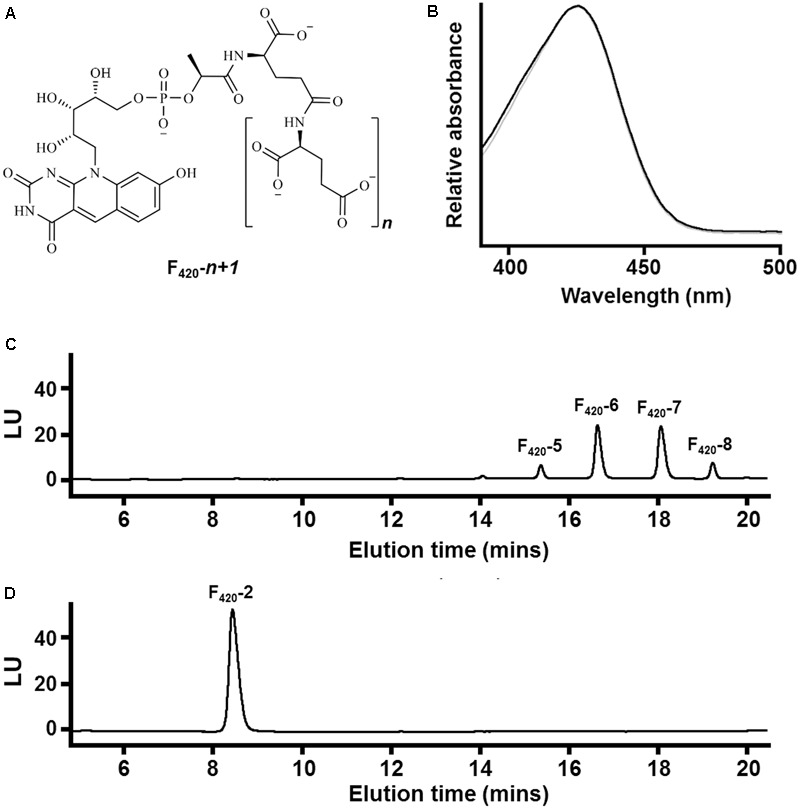
Chemical composition of F_420_ purified from different sources. **(A)** Chemical structure of F_420_ showing the redox-active isoalloxazine headgroup and lactyloligoglutamyl tail. The number of glutamate residues (*n*) varies between bacteria and archaea. **(B)** Absorbance spectrum of F_420_ purified from *Mycobacterium smegmatis* mc^2^4517 (black) and *Methanothermobacter marburgensis* A60 (gray). **(C)** HPLC trace showing F_420_ purified from *M. smegmatis* mc^2^4517 varies in chain length between five to eight glutamates. **(D)** HPLC trace showing F_420_ purified from *Mtb. marburgensis* A60 predominantly contains two glutamate residues.

Nevertheless, there remains an incomplete understanding of how the chemical structure of F_420_ relates to its physiological function and industrial application. Structurally, the cofactor comprises two major components (**Figure 1A**): (i) a redox-active headgroup comprising a modified isoalloxazine tricycle and (ii) a catalytically-inactive side chain comprising a ribitylphospholactyl moiety and an oligoglutamate chain of variable length (Eirich et al., 1978; Ashton et al., 1979). F_o_ (8-hydroxy-5-deazaflavin), a chromophore used by DNA photolyases, serves as the biosynthetic precursor to F_420_ (Graupner and White, 2001). The phospholactyl and oligoglutamate constituents are added to this precursor by three dedicated biosynthetic enzymes (CofC, CofD, CofE) (Nocek et al., 2007; Forouhar et al., 2008; Grochowski et al., 2008; Bashiri et al., 2016). It is well-established that key chemical substitutions in the isoalloxazine group confer the unique redox properties of deazaflavins over flavins (Walsh, 1986; Greening et al., 2016). However, it remains to be understood why organisms have selected to incorporate the lactyloligoglutamate side chain. It also remains elusive why the length of the oligoglutamate chain varies between organisms: two to three residues in methanogens without cytochromes, three to six in Proteobacteria and methanogens with cytochromes (Methanosarcinales), and five to eight in Actinobacteria and Chloroflexi (Gorris and van der Drift, 1994; Bair et al., 2001; Ney et al., 2017). The side chain does not significantly affect the chemical reactivity or redox properties of F_420_ relative to its precursor F_o_ (Greening et al., 2016); indeed, previous studies have shown that the redox potential of F_420_ is the same as F_o_ (-340 mV) and hence is not modulated by the oligoglutamate tail (Jacobson and Walsh, 1984). Moreover, while the charged nature of F_420_ ensures it does not diffuse from the cell in contrast to its precursor F_o_ (Ney et al., 2017), this does not explain why organisms selected to synthesize a polyanionic rather than monoanionic cofactor.

We recently hypothesized that catalytic constraints may have driven the synthesis of the side chain in F_420_ (Ney et al., 2017). Specifically, the oligoglutamate chain may facilitate higher-affinity electrostatic interactions between enzyme and cofactor. We propose that, in addition to driving specific F_420_-dependent reactions, such high-affinity interactions may be crucial for maintaining redox homeostasis and discriminating between cofactor pools (Ney et al., 2017). In support of this observation, two cofactor-bound crystal structures suggest that the oligoglutamate chain can interact with surface cationic residues of F_420_-dependent oxidoreductases (Cellitti et al., 2012; Ahmed et al., 2016), though the significance of this has not been considered. Other structural analyses have proposed that, while the ribityl and phosphate groups of F_420_ make hydrogen bonds with surrounding residues, the oligoglutamate tail instead extends into the solvent phase without contributing to binding (Bashiri et al., 2008). In this work, we addressed the effect of the oligoglutamate side chain on the catalytic activity of F_420_-dependent oxidoreductases. To do this, we purified F_420_ from two sources: short-chain F_420_ from a methanogen and long-chain F_420_ from a mycobacterium. We subsequently studied the cofactor binding affinities and substrate consumption kinetics of mycobacterial F_420_-dependent oxidoreductases in the presence of these different F_420_ variants. We focused on a representative from each of the three main superfamilies of F_420_-dependent oxidoreductases found in bacteria (Selengut and Haft, 2010), namely the luciferase-like hydride transferases (LLHTs; TIM barrel fold) (Bashiri et al., 2008; Greening et al., 2016) and flavin/deazaflavin oxidoreductase superfamilies A (FDOR-As; monomeric split β-barrel proteins) and B (FDOR-Bs; dimeric split β-barrel proteins) (Ahmed et al., 2015; Greening et al., 2016).

## Materials and Methods

### F_420_ Production

Long-chain F_420_ was recombinantly overproduced in *Mycobacterium smegmatis* mc^2^4517 cells harboring an inducible pYUBDuet shuttle vector encoding the F_420_ biosynthesis genes *cofC, cofD*, and *cofE* (Bashiri et al., 2010). Cultures were grown in twenty 2 L Erlenmeyer flasks each containing 500 mL LB broth supplemented with 0.05% Tween 80 (LBT), 50 μg mL^-1^ hygromycin B and 20 μg mL^-1^ kanamycin. The cultures were grown to stationary-phase in a rotary incubator (200 rpm) at 37°C for 5 days before harvesting. Short-chain F_420_ was extracted from a thermophilic methanogen strain, *Methanothermobacter marburgensis* A60. We isolated the strain by repeated serial dilution of geothermally heated sediments from Ngatamariki, New Zealand. 16S rRNA gene sequencing of genomic DNA extracts (NucleoSpin Tissue Kit, Macherey-Nagel) using the archaeal-specific primer set 109f/912r confirmed the strain shared 99% sequence identity with the well-studied laboratory strain *Methanothermobacter marburgensis* Marburg^T^ (Liesegang et al., 2010). For F_420_ production, the strain was cultured in thirty 1 L bottles each containing 400 mL of a previously defined media supplemented with 29 mM sodium formate (Sparling et al., 1993) and a H_2_/CO_2_ atmosphere (80:20 v/v). Cultures were grown to stationary-phase in a rotary incubator (100 rpm) at 60°C for 3 days with periodic gas feeding before harvesting.

### F_420_ Purification

F_420_ was harvested from the mycobacterial and methanogen cultures through variations on an existing protocol (Isabelle et al., 2002). The cells were harvested by centrifugation at 10,000 × *g* for 20 min, the resultant pellets were washed, and the cultures were resuspended in 20 mM TrisHCl (pH 7.5) at a ratio of 1 g per 10 mL. The cells were autoclaved at 121°C to release F_420_, a heat-stable cofactor, into the buffer. The cell debris was removed by centrifugation at 18,000 × *g* for 20 min and the supernatant was decanted and vacuum-filtrated through 0.45 μm filter paper. The F_420_ was isolated by FPLC (fast protein liquid chromatography) with a Macro-prep High Q Resin anion exchange column (Bio-Rad). A gradient of buffer A (20 mM TrisHCl, 100 mM NaCl, pH 7.5) and buffer B (20 mM TrisHCl, 1 M NaCl, pH 7.5) was applied, with buffer B increasing from 0 to 100% over 10 column volumes. Fractions containing F_420_ were identified *via* analysis of absorbance spectra on a SpectraMax^®^ M3 Multi-Mode Microplate Reader (Bio-Strategy, Australia). Fractions containing F_420_ were pooled. The F_420_ solution was further purified and concentrated by hydrophobic interaction chromatography through a high capacity C18 column equilibrated in H_2_O. F_420_ was eluted in 2 mL fractions in 20% methanol, dried by rotary evaporation, and stored at -20°C.

### HPLC and LC/MS Analysis

An ion-paired reverse phase HPLC (high-performance liquid chromatography) protocol was used to determine the oligoglutamate chain length of F_420_ purified from mycobacterial and methanogen sources. An Agilent 1200 series system equipped with an Agilent Poroshell 120 EC-C18 2.1 × 50 mm 2.7 μm column and diode array detector was used. The F_420_ species were separated at a flow rate of 0.3 mL min^-1^ using a gradient of two buffers, namely A (20 mM ammonium phosphate, 10 mM tetrabutylammonium phosphate, pH 7.0) and B (100% acetonitrile). A gradient was run from 25 to 40% buffer B as follows: 0–1 min 25%, 1–10 min 25–35%, 10–13 min 35%, 13–16 min 35–40%, 16–19 min 40–25%. F_420_ absorbance was measured at 420 nm using a diode array detector. This system was also used to execute an absorbance scan from 400 to 600 nm on the sole F_420_-2 peak of the methanogen F_420_ and the prominent F_420_-6 peak of the mycobacterial F_420_. The length of the F_420_ oligoglutamate tails were verified by a reverse phase LC/MS (liquid chromatography / mass spectrometry) protocol with an Agilent 1100 series LC/MSD TOF equipped with a Poroshell 120 EC-C18 2.1 × 100 mm 2.7 μm column. A gradient protocol comprising of Buffer A (20 mM ammonium acetate pH 6.8) and Buffer B (100% acetonitrile) was applied as follows: Held from 0 – 1 min at 5% B; 1 – 10 min from 5 – 20% B. Negative mode ESI was used with a capillary voltage of 2500 V and gas temperature of 300°C. The system was run at a flow rate of 0.2 mL min^-1^ and chemical species were scanned from 150 – 1500 m/z.

### Enzymatic Assays

The F_420_-reducing glucose 6-phosphate dehydrogenase (Fgd; MSMEG locus 0777) and two F_420_H_2_-dependent reductases (FDORs; MSMEG loci 2027, 3380) from *M. smegmatis* mc^2^155 were recombinantly overexpressed in *Escherichia coli* BL21(DE3) using previously described vectors and protocols (Taylor et al., 2010; Ahmed et al., 2015). Cells were harvested by centrifugation, resuspended in lysis buffer, and lysed in an EmulsiFlex-C3 homogenizer (ATA Scientific, Australia) according to previously described protocols (Greening et al., 2017). Enzymes were purified from soluble extracts by Ni-nitrilotriacetic acid (NTA) affinity chromatography using gravity columns as previously described (Taylor et al., 2010; Ahmed et al., 2015) and stored in elution buffer (50 mM NaH_2_PO_4_ 300 mM NaCl, 250 mM imidazole, pH 8.0) until use in assays. The high purity of the proteins was confirmed by running the fractions on NuPAGE Novex 10% Bis-Tris gels (Invitrogen, Australia) and staining with Coomassie Brilliant Blue. We measured the activities of the enzymes by monitoring the rates of F_420_ reduction or F_420_H_2_ oxidation in the presence of different substrate concentrations; this serves as a reliable measure of substrate transformation given F_420_-dependent oxidoreductases directly mediate hydride transfer between cofactor and substrate in an equimolar manner (Greening et al., 2016; Jirapanjawat et al., 2016). Enzyme activities were measured in 96-well plates containing degassed TrisHCl buffer [200 mM TrisHCl, 0.1% (w/v) Triton X-100, pH 8.0] sequentially supplemented with substrate at the specified concentration, 50 μM of the relevant cofactor, and 100 nM of the relevant enzyme. Reaction rates were measured by monitoring the initial linear change of absorbance of the reaction mixture at 420 nm using a SpectraMax^®^ M3 Multi-Mode Microplate Reader (Molecular Devices); loss of absorbance was observed due to Fgd-mediated reduction of F_420_, whereas gain of absorbance occurred due to FDOR-mediated reoxidation of F_420_H_2_. Prior to measurement of FDOR activity, F_420_ was enzymatically reduced with 1 μM Fgd in a nitrogen glovebox for 4 h and purified by spin filtration as previously described (Ahmed et al., 2015). Reaction velocities were calculated by subtracting rates of no-enzyme controls from the initial linear rates of F_420_ reduction or F_420_H_2_ reoxidation measured.

### Intrinsic Tryptophan Fluorescence Quenching

F_420_ dissociation constants were calculated by monitoring the decrease of intrinsic tryptophan fluorescence upon gradual titration of F_420_ as previously described (Ahmed et al., 2015). A SpectraMax^®^ M3 Multi-Mode Microplate Reader (Molecular Devices) with a quartz cuvette containing 500 nM of protein in 20 mM TrisHCl, pH 8.0 at 24°C was used. Samples were excited at 290 nm and emission was monitored at 340 nm. One microliter aliquots of F_420_ standards in the same buffer were added to produce a solution with final concentrations ranging from 0 to 12.3 μM F_420_, with the concentration recalculated for the incremental increase in volume. The fractional saturation (F/F_max_) was plotted against the concentration of free F_420_, and the *K*_d_ derived from fitting the data points to the function: F/F_max_ = F_max_^∗^[Free F_420_] / (*K*_d_ + [Free F_420_]).

### Molecular Dynamics Simulations

Molecular dynamics simulations used the 1.5 Å resolution crystal structure of MSMEG_2027 [PDB: 4Y91 (Ahmed et al., 2015)] and 1.2 Å resolution structure of MSMEG_3380 [PDB: 3F7E (Taylor et al., 2010)]. The structure of the 26 residues missing from the MSMEG_2027 crystal structure was predicted by homology modeling in Phyre2 (intensive mode) (Kelley et al., 2015) using *M. tuberculosis* Rv3547/Ddn [PDB: 3RZ (Cellitti et al., 2012)] as the template. Simulations were visualized in the VMD: Visual Molecular Dynamics software (Humphrey et al., 1996) and calculations were performed using Amber16 (University of California San Francisco) employing the ff14SB forcefield (Maier et al., 2015). The Antechamber module within Amber16 was used to parameterize the F_420_-2 and F_420_-6 moieties with the GAFF2 forcefield and mulliken charges, and ionsjc_TIP3P parameters were used for the Na^+^ counterions. F_420_-2 and F_420_-6 were modeled into the structure of MSMEG_3380 and docked into the structure of MSMEG_2027 using AutoDock Vina (Morris et al., 2009). For MSMEG_3380, the positions of the cofactor up to the first glutamate residue were based on the cofactor position in the homologous protein Rv1155 [PDB: 4QVB (Mashalidis et al., 2015)]. Diglutamate and hexaglutamate tails were manually constructed and initial geometry optimisations were performed in Discovery Studio 3.5 (Accelrys). The headgroup was constructed in its deprotonated F_420_H^-^ form (Mohamed et al., 2016a) and all carboxylate groups were modeled in deprotonated form. Protein-cofactor complexes were solvated in an octahedral TIP3P water box with a minimum periodic boundary distance of 10.0 Å from the solute. Each system was relaxed for a maximum of 25,000 steps of steepest descent and 25,000 steps of conjugate gradient whilst constraining the protein atoms, after which a production run of 400 ns was performed at 298 K and 1 bar with a pressure relaxation time of 2.0 ps. Langevin dynamics was employed with a collision frequency of 5.0 and SHAKE constraints were applied to all hydrogen atoms. The ribityl-bearing nitrogen of the headgroup of each F_420_ moiety had light positional restraints enforced (10 kcal mol^-1^ Å^2^) to prevent the headgroup from leaving the active site and to allow maximal rotational freedom within the active site so as minimize bias on the interaction energy. MMPBSA (Molecular Mechanics Poisson Boltzmann Surface Area) calculations were carried out on 2,000 frames of each 400 ns simulation employing an ionic strength of 0.15 mM and fillratio setting of 4.0.

### Comparative Structural Analysis

For comparative structural analysis, protein sequences of F_420_-dependent oxidoreductases from different subgroups within the LLHT, FDOR-A, and FDOR-B superfamilies were retrieved from the NCBI database. Multiple sequence alignments were constructed with Clustal Omega (Sievers et al., 2011). Homology models of MSMEG_0777 and Rv0132c were constructed in RaptorX (Källberg et al., 2012) using *M. tuberculosis* Rv0407/Fgd as the template (Bashiri et al., 2008). Protein structures were visualized in UCSF Chimera (Pettersen et al., 2004).

## Results

### The F_420_ Oligoglutamate Chain Influences Cofactor-Binding Affinity and Reaction Kinetics of F_420_-Dependent Oxidoreductases

At present, no chemical syntheses or enzymatic cascades have been developed for cell-free production of F_420_. We therefore obtained sufficient F_420_ for this study through large-scale cultivation of two F_420_-producing strains, namely the new methanogen isolate *Methanothermobacter marburgensis* A60 and a previously described F_420_ overproduction strain of *Mycobacterium smegmatis* mc^2^4517 (Bashiri et al., 2010), under conditions that would promote high-level F_420_ production. F_420_ was purified from these strains through a sequence of anion-exchange chromatography, hydrophobic interaction chromatography, and rotary evaporation. We detected the eponymous absorbance peak of F_420_ in the purified fractions (**Figure 1B**). To confirm chain length, we separated the purified F_420_ on a HPLC equipped with an anion-exchange column and detected the cofactor at 420 nm using a diode array detector. F_420_ purified from *M. smegmatis* contained between five to eight glutamates (**Figure 1C**), consistent with previous mass validation (Bashiri et al., 2010; Ney et al., 2017). In contrast, HPLC traces showed that all detectable F_420_ purified from *Mtb. marburgensis* contained two glutamate residues (**Figure 1D**). The mass of the dominant chemical species was validated by LC/MS (Supplementary Figure S1).

**FIGURE 2:**
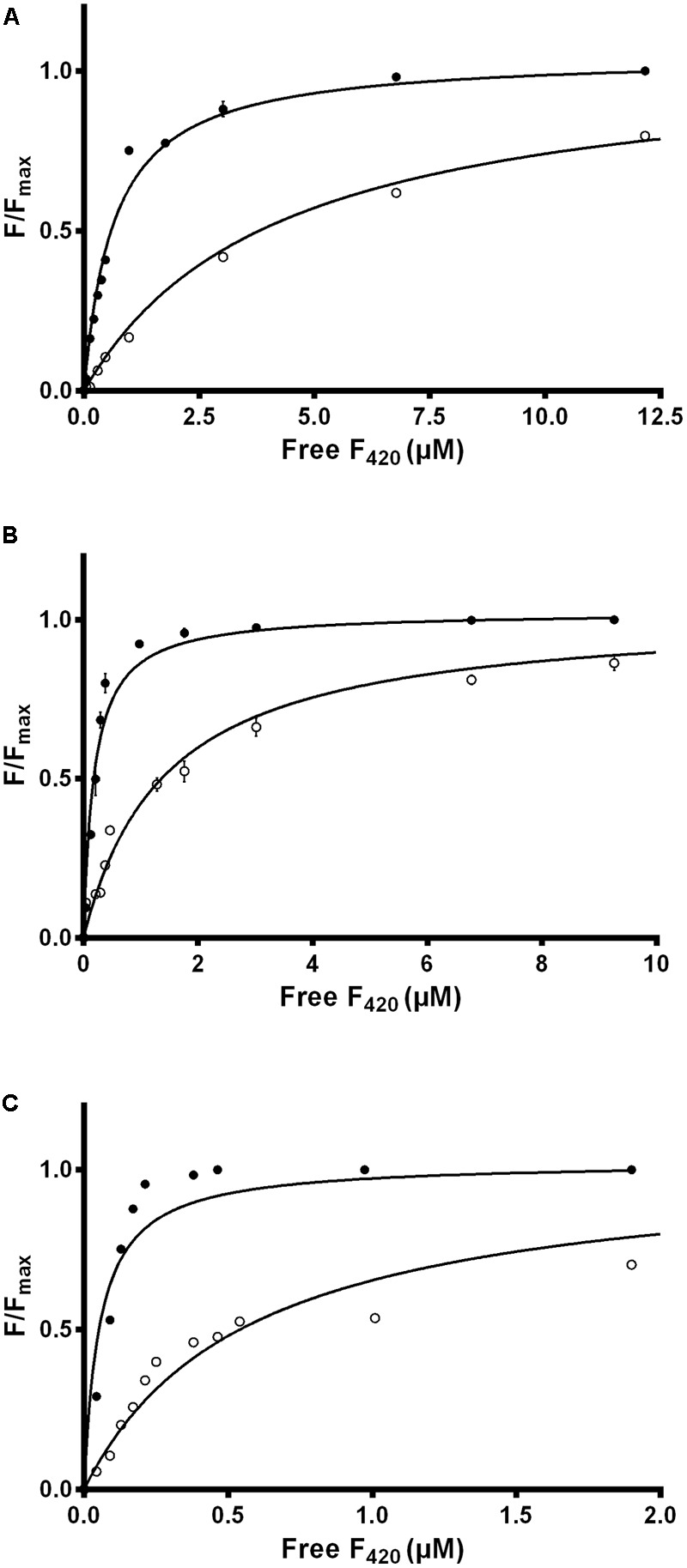
Affinity of F_420_ for F_420_-dependent oxidoreductases depends on oligoglutamate chain length. The intrinsic fluorescence quenching caused by the binding of the cofactor was measured with the **(A)** F_420_-dependent glucose 6-phosphate dehydrogenase MSMEG_0777, **(B)** F_420_H_2_-dependent reductase MSMEG_2027, and **(C)** F_420_H_2_-dependent reductase MSMEG_3380. Quenching is shown with long-chain mycobacterial F_420_ (●) and short-chain methanogen F_420_ (○). Supplementary Figure S2 shows additional data points for the methanogen F_420_ that are omitted here. Error bars show standard deviations from three independent replicates.

We used intrinsic fluorescence quenching to determine the binding affinities of the two purified F_420_ variants for three F_420_-dependent oxidoreductases from *M. smegmatis*: the F_420_-dependent glucose 6-phosphate dehydrogenase MSMEG_0777 (LLHT family), the F_420_H_2_-dependent quinone reductase MSMEG_2027 (FDOR-A family), and a promiscuous F_420_H_2_-dependent reductase of unknown function MSMEG_3380 (FDOR-B family). We observed that long-chain mycobacterial F_420_ bound the enzymes with nanomolar affinities (*K*_d_ values) of 650 nM, 190 nM, and 54 nM respectively (**Figure 2** and Supplementary Figure S2), similar to values derived from previous enzymatic studies (Bashiri et al., 2008; Ahmed et al., 2015). In contrast, short-chain methanogen F_420_ bound with six- to ten-fold lower affinities, i.e., 4.1 μM, 1.4 μM, and 570 nM respectively (**Figure 2** and Supplementary Figure S2). We observed no significant binding of the biosynthetic precursor F_o_, at concentrations up to 50 μM, for any of the three enzymes. This finding suggests that interactions between the F_420_ oligoglutamate chain and mycobacterial F_420_-dependent oxidoreductases are crucial for high-affinity cofactor-enzyme associations.

**FIGURE 3:**
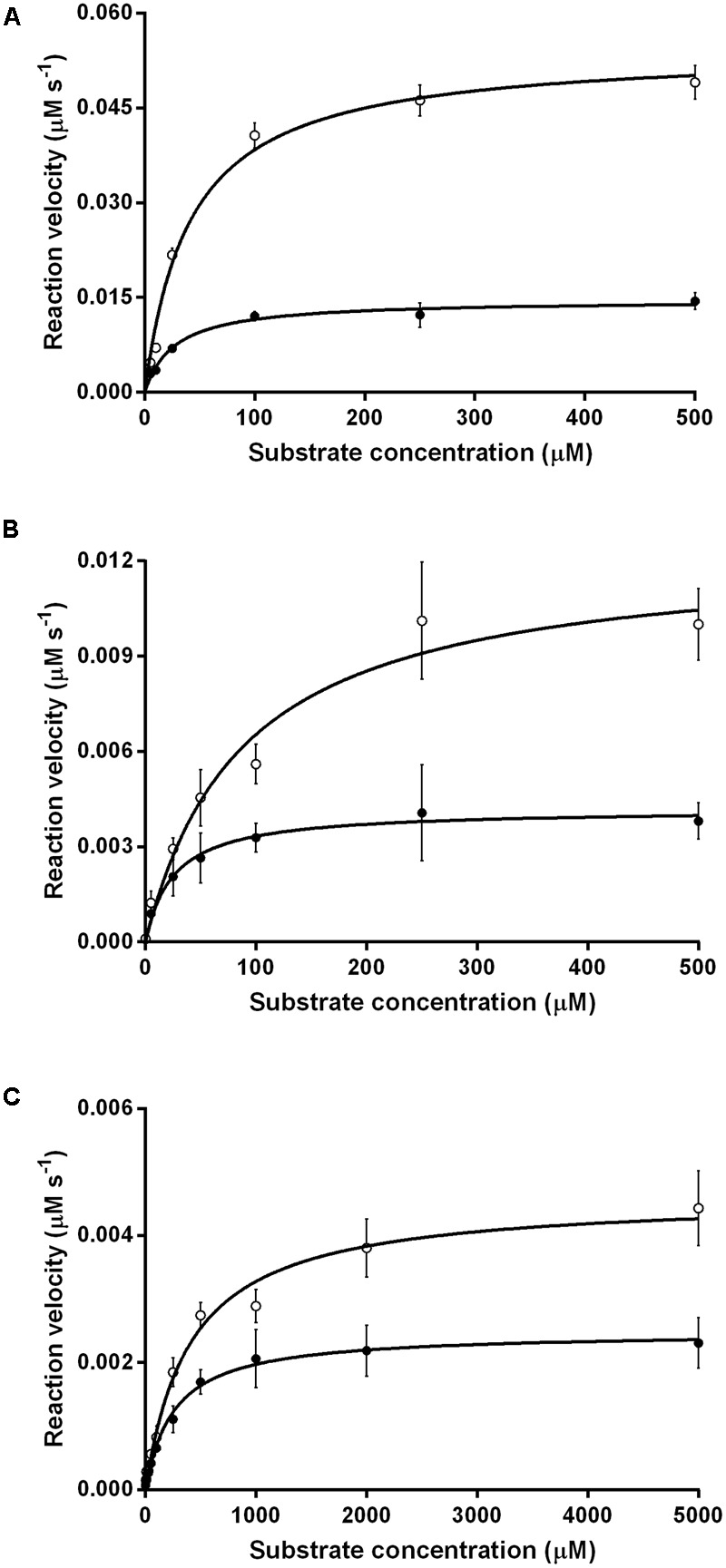
Kinetics of substrate oxidation/reduction of F_420_-dependent oxidoreductases depends on F_420_ oligoglutamate chain length. Three activities were measured, namely **(A)** F_420_-dependent oxidation of glucose 6-phosphate by native Fgd activity, **(B)** F_420_H_2_-dependent reduction of menadione by native MSMEG_2027 activity, and **(C)** F_420_H_2_-dependent reduction of cyclohexenone by promiscuous MSMEG_3380. Activities are shown with long-chain bacterial F_420_ (●) and short-chain methanogen F_420_ (○). Error bars show standard deviations from three independent replicates.

We compared the reaction kinetics of the three enzymes in the presence of the different F_420_ variants. The enzymes were catalytically active in the presence of F_420_ purified from both sources. Consistent with previous findings (Bashiri et al., 2008; Taylor et al., 2010; Ahmed et al., 2015), reaction kinetics of all three enzymes followed Michaelis–Menten models (**Figure 3**), though the kinetic parameters differed depending on the length of the F_420_ oligoglutamate chain. Observed substrate turnover rates (*k*_cat_) were between 1.9 and 3.7 times greater in the presence of short-chain F_420_ compared to long-chain F_420_ (**Table 1**); such enhancements of initial rate were observed irrespective of the F_420_ concentration used (Supplementary Figure S3). A decrease in *K*_m_ was also observed in the presence of the long-chain species, with differences ranging from 3.5-fold for MSMEG_2027 to a modest 1.5-fold for MSMEG_0777 and MSMEG_3380 (**Table 1**). Hence, high-affinity cofactor binding results in both enhanced substrate binding and decreased reaction turnover.

**Table 1:** Kinetic parameters of F_420_-dependent oxidoreductases in the presence of long-chain F_420_ and short-chain F_420_.

Enzyme	F_420_ *K*_d_ (μM)	*K*_M_ (μM)	*K*_cat_ (s^-1^)	*K*_cat_/*K*_M_ (M^-1^ s^-1^)
**Fgd: Glucose 6-phosphate oxidation**				
F_420_-long	0.65 ± 0.05	26.8 ± 4.4	0.292 ± 0.012	10900
F_420_-short	4.18 ± 0.29	41.3 ± 4.3	1.086 ± 0.029	26300
**MSMEG_2027: Menadione reduction**				
F_420_-long	0.19 ± 0.02	25.6 ± 9.2	0.084 ± 0.007	3280
F_420_-short	1.43 ± 0.12	88.2 ± 18.4	0.244 ± 0.018	2770
**MSMEG_3380: Cyclohexenone reduction**				
F_420_-long	0.054 ± 0.010	259 ± 41	0.025 ± 0.001	96
F_420_-short	0.57 ± 0.05	407 ± 52	0.046 ± 0.002	177

*The dissociation constants (*K*_*d*_) of the different F_*420*_ species is shown in the second column. The kinetic parameters of substrate oxidation or reduction by the enzymes is shown in the subsequent four columns. Error margins show standard deviations from three independent replicates.*

### The Oligoglutamate Chain Makes Multiple Electrostatic Interactions with Surface Anionic Residues of FDORs

**FIGURE 4:**
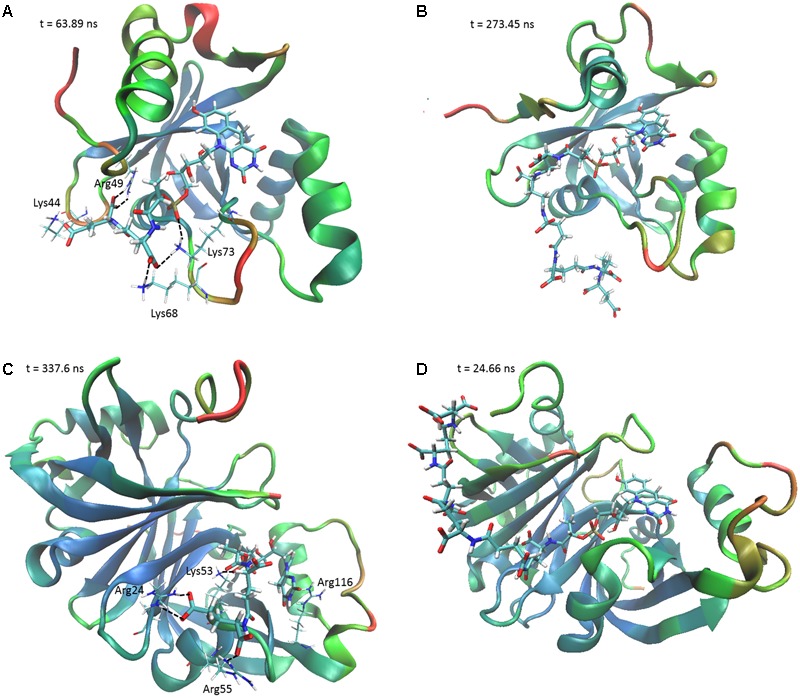
The F_420_ oligoglutamate chain makes electrostatic interactions with FDORs. Snapshots are shown of the molecular dynamics simulations of **(A)** MSMEG_2027 with F_420_-2 (*t* = 63.89 ns), **(B)** MSMEG_2027 with F_420_-6 (*t* = 273.45 ns), **(C)** MSMEG_3380 with F_420_-2 (*t* = 337.6 ns), and **(D)** MSMEG_3380 with F_420_-6 (*t* = 24.66 ns) with some of the more stable interactions observed. The colors on the secondary structure of the enzyme represent flexibility as calculated by the backbone RMSD over the course of the whole simulation, with red representing high mobility and blue representing high stability. Black dashed lines indicate electrostatic interactions. The cofactor is depicted in stick representation with thicker bonds, whereas the relevant residues are depicted as sticks with thinner bonds. Interacting residues are not shown for F_420_-6 for clarity.

To determine the structural and mechanistic basis for these differences, we used molecular dynamics simulations to compare the binding of long-chain (F_420_-6) and short-chain (F_420_-2) variants of F_420_ to the available crystal structures of the FDOR-A MSMEG_2027 (Ahmed et al., 2015) and the FDOR-B MSMEG_3380 (Taylor et al., 2010). An overarching characteristic of all simulations was that, following equilibration of the position of the cofactor tail, the glutamate residues made multiple transient electrostatic contacts with specific arginine and lysine residues on the surfaces of the oxidoreductases. The first two glutamates of both F_420_-2 and F_420_-6 made interactions with residues Lys73, Arg49, Lys44, and sporadically Lys68 of MSMEG_2027 (**Figures 4A,B**). These glutamate residues also interacted with Arg205, Arg23, and Arg54 of MSMEG_3380, but these interactions were more transient (**Figures 4C,D**). In both cases, the terminal four glutamates of F_420_-6 made multiple transient electrostatic interactions with surface cationic residues, but these residues were highly dynamic and displayed little specificity in the various interactions they formed. These additional interactions also appeared to stabilize the core interactions made by the cofactor, for example between the phosphate group and Lys53 of MSMEG_3380 (**Figures 4B,D**). The multiple alternative tail conformations observed remained favorable even though the headgroup was weakly restrained within the active site (Supplementary Figure S4).

**FIGURE 5:**
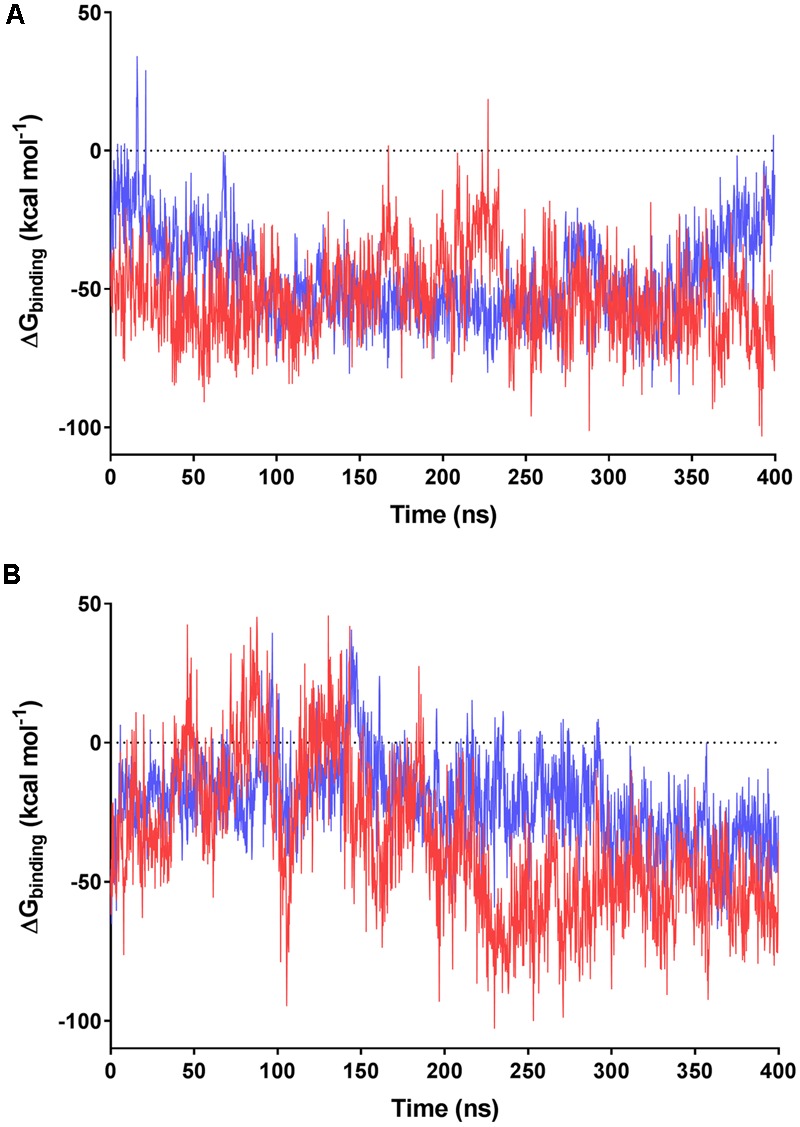
MMPBSA-calculated binding energies of F_420_ variants with F_420_-dependent oxidoreductases. Data based on the 400 ns trajectories for the molecular dynamics simulations with **(A)** MSMEG_2027 and **(B)** MSMEG_3380. The calculated binding energies are shown for F_420_-2 in blue and F_420_-6 in red.

We subsequently evaluated the cofactor-enzyme binding energies for the trajectories using MMPBSA analysis, which confirmed that the oligoglutamate chain modulated binding. While the cofactor-enzyme contacts reduced solvation energy, this was offset by the combined energies from 1 to 4 electrostatic and other non-bonded interactions, resulting in a lower total ΔG_binding_ for the complex. Consistent with the additional dynamic interactions observed, F_420_-6 reached lower ΔG_binding_ values than F_420_-2 in simulations with both MSMEG_2027 and MSMEG_3380 (**Figures 5A,B**). Such observations are consistent with the lower *K*_d_ values determined in the tryptophan fluorescence quenching experiments (**Figure 2** and Supplementary Figure S2). Loss of multiple electrostatic interactions between the enzyme and cofactor tail increased the solvation energy and often pushed ΔG_binding_ into positive territory (**Figure 5B**). The internal bond, angle, and dihedral energies of the cofactor were essentially unaffected by the various binding modes (Supplementary Figure S4), emphasizing that most binding energy changes occur due to flexibility of the oligoglutamate chain rather than the isoalloxazine ring. These findings in turn suggests that there are no combinations of interactions that would release the headgroup from the active site and instead cofactor dissociation may be driven by tail solvation.

Interestingly, we observed that the monomeric enzyme MSMEG_2027 exhibited markedly different cofactor binding modes compared to the dimeric enzyme MSMEG_3380. While most of the interacting positive charges were accommodated on the flexible loop regions of MSMEG_2027 (Lys44, Lys68, Lys73), the main contacting residues of MSMEG_3380 (Arg205, Arg23, Arg54) all lie on highly stable β-sheet and helical secondary structural regions. Multiple sequence alignments and comparative structural analysis suggest that these cationic residues were highly conserved within their respective FDOR-A and FDOR-B superfamilies (Supplementary Figures S5, S6). Most notably, the sequence motif Gx[KR]xG[QKE]xR occurs in all enzymes in the FDOR-A superfamily, among them enzymes sharing less than 25% identity. This sequence forms a loop joining two β-strands (Ahmed et al., 2015); the Gly residues likely contribute to the flexibility of the loop and the cationic residues serve as the main site of sustained interaction with the oligoglutamate chain (**Figure 4**). Conserved cationic surface residues are also proximal to the F_420_ oligoglutamate chain in the LLHT superfamily (Supplementary Figures S5, S6), providing further support that electrostatic interactions generally occur between bacterial F_420_-dependent oxidoreductases and the F_420_ oligoglutamate chain.

## Discussion

Since the structure of F_420_ was proposed in Eirich et al. (1978), studies on its catalytic behavior have focused on its redox-active headgroup (Walsh, 1986; Greening et al., 2016) and the role of its side chain has not been addressed. In this study, we reveal that the F_420_ oligoglutamate chain modulates catalysis in bacterial F_420_-dependent oxidoreductases. Our experimental findings demonstrate that synthesis of long-chain F_420_ results in higher-affinity enzyme-cofactor interactions. Molecular dynamics simulations focused on FDOR-A and FDOR-B representatives provide a rationale for these findings by showing that the F_420_ tail electrostatically interacts with conserved cationic residues on the surface of mycobacterial F_420_-dependent oxidoreductases; while the diglutamate chain can make sustained electrostatic interactions, the multiple additional transient interactions made by oligoglutamate chain offsets solvation energy and increases binding energy. We made compatible findings across three different protein families in the presence of both physiological and non-physiological substrates. It is therefore probable that the oligoglutamate chain of F_420_ is of general relevance to catalysis of bacterial F_420_-dependent oxidoreductases.

We also observed that higher-affinity cofactor binding modulates reaction kinetics by increasing substrate affinity but decreasing turnover. Such findings likely reflect that mycobacterial F_420_-dependent oxidoreductases mediate catalysis through a ternary complex, with hydride transfer occurring directly between the isoalloxazine headgroup of the cofactor and the substrate (Greening et al., 2016; Mohamed et al., 2016b). If cofactor dissociation is the rate-limiting step in the catalytic cycle of these oxidoreductases, higher-affinity cofactor-enzyme interactions may result in lower cofactor dissociation rates (*k*_off_) and hence reduced substrate turnover. It is also plausible that conformational changes caused by the electrostatic interactions are transmitted to the adjoined isoalloxazine- and substrate-binding sites, thereby modulating substrate affinity; this may be particularly important in FDOR-A proteins, where interactions between the terminal glutamate residues and the lysine-rich loop region may stabilize the split β-barrel fold and in turn the substrate-binding site. The finding that substrate turnover of F_420_-dependent oxidoreductases is accelerated in the presence of short-chain F_420_ is important for biotechnological reasons: it suggests that F_420_ purified from methanogen sources will result in higher turnovers in the various bioremediation and biocatalysis processes for which F_420_-dependent oxidoreductases have been advocated (Taylor et al., 2013; Greening et al., 2016, 2017). A tradeoff would be the reduction in substrate affinity, but this is likely to be negligible for biocatalytic applications given they rely on high substrate concentrations. Obstacles in metabolic engineering must be overcome, however, if short-chain F_420_ is to be heterologously produced at industrially-relevant scales.

Future studies are required to determine whether the observed tradeoffs between affinity and turnover are physiologically relevant. We hypothesize that the oligoglutamate chain ensures the affinity of interactions between cofactor and enzyme remain in the physiologically desirable nanomolar range. In turn, this may increase the substrate specificity of the oxidoreductases that bind the cofactor. In bacterial cells, loss of this chain may compromise specific F_420_-dependent reactions and have wider effects on redox homeostasis and cofactor partitioning. Consistently, studies on nitroimidazole resistance suggest that the enzyme responsible for oligoglutamate chain elongation, CofE (F_420_-0:γ-glutamyl ligase), is required for optimal functionality of F_420_ in mycobacterial cells, though is less important than the other F_420_ biosynthetic enzymes (Haver et al., 2015). In contrast, most methanogens appear to suffice with a diglutamate- rather than oligoglutamate-containing side chain. One explanation is that F_420_-dependent enzymes in such organisms may be less kinetically constrained, given F_420_ serves as the primary catabolic cofactor and is generally present at higher concentrations than in bacterial cells (Thauer et al., 2008). However, further studies are required to understand the significance of the F_420_ diglutamate chain in the catalysis of F_420_-dependent oxidoreductases in methanogens and why Methanosarcinales synthesize longer-chain F_420_ variants (Gorris and van der Drift, 1994).

The observed differences between bacterial and archaeal F_420_ may also be relevant for understanding the evolution of the biosynthesis of deazaflavins. The F_420_ biosynthesis pathway appears to have undergone a complex evolutionary trajectory, with phylogenetic evidence unable to resolve whether the cofactor originated in bacteria, archaea, or the last universal common ancestor (Nelson-Sathi et al., 2015; Weiss et al., 2016; Ney et al., 2017). We recently proposed that F_o_ served as the primordial cofactor in deazaflavin-dependent enzymes, but selective pressure to produce a membrane-impermeable derivative resulted in the evolution of F_420_ biosynthetic enzymes (CofC, CofD, CofE) and in turn the production of short-chain F_420_ (Ney et al., 2017). We propose here that the synthesis of longer-chain derivatives was driven by selection pressure for higher-affinity cofactor-enzyme interactions or more controlled redox homeostasis. This was likely mediated through evolution of the CofE, which is a single-domain enzyme in short-chain F_420_ producers but is fused with an FMN reductase domain in most long-chain producers (Ney et al., 2017); recent structural and kinetic studies on mycobacterial CofE have demonstrated this second domain is essential for elongation of the oligoglutamate chain (Bashiri et al., 2016). It is plausible that the three families of bacterial F_420_-dependent oxidoreductases co-evolved with CofE, resulting in higher-affinity cofactor-enzyme interactions.

## Author Contributions

CG and AW conceived the study. CG, AW, BN, CC, RS, TJ, JO, and MS designed experiments. BN, TJ, AW, CG, CC, RS, and MS performed experiments. CG and CJ supervised students. CG, AW, BN, JO, TJ, and CJ interpreted data. CG, AW, and BN wrote the paper.

## Conflict of Interest Statement

The authors declare that the research was conducted in the absence of any commercial or financial relationships that could be construed as a potential conflict of interest.
