# Supplementary material for: Cofactor Tail Length Modulates Catalysis of Bacterial F420-Dependent Oxidoreductases
Source: Front Microbiol. 2017 Sep 27;8:1902. doi: 10.3389/fmicb.2017.01902 (PMC5623714; doi:10.3389/fmicb.2017.01902)
Supplement: Supplementary file 1 [file Data_Sheet_1.DOCX]

**Supporting Information**

**Figure S1.** Mass spectrum of F_420_ purified from *Methanothermobacter marburgensis* A60. The dominant peak corresponds to the molecular weight of F_420_-2. A mass spectrum of F_420_ purified from *Mycobacterium smegmatis* mc^2^4517 was previously published (Ney et al., 2017).


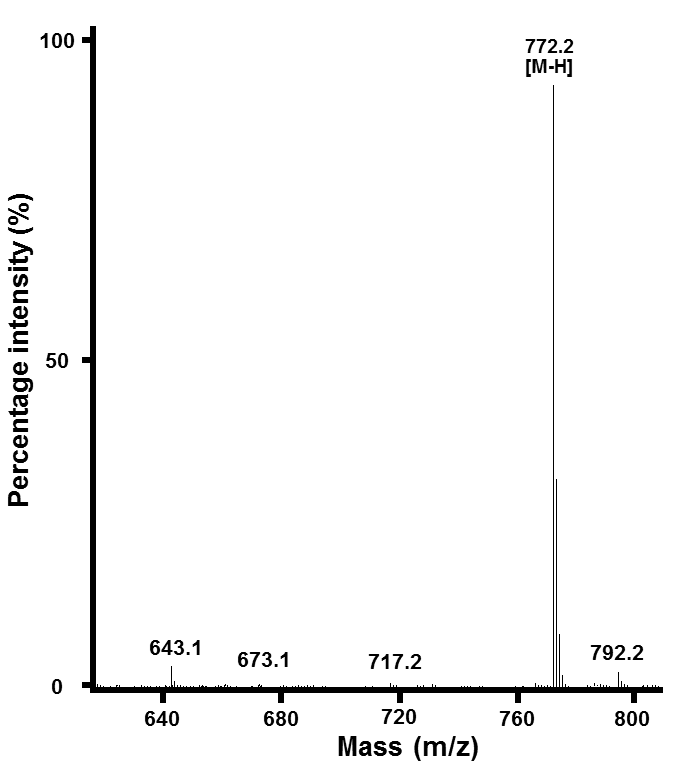


**Figure S2.** Complete results of differences in intrinsic tryptophan quenching in the presence of F_420_ of different oligoglutamate chain lengths. Binding of the cofactor to the (A) F_420_-dependent glucose 6-phosphate dehydrogenase MSMEG_0777, (B) F_420_H_2_-dependent reductase MSMEG_2027, and (C) F_420_H_2_-dependent reductase MSMEG_3380 were measured. Quenching is shown with long-chain mycobacterial F_420_ (●) and short-chain methanogen F_420_ (○).





**Figure S3.** Differences in kinetics of substrate oxidation by F_420_-dependent glucose 6-phosphate dehydrogenase in the presence of different concentrations of long-chain mycobacterial F_420_ (light bars) and short-chain methanogen F_420_ (dark bars). In all cases, the initial substrate concentration was 50 μM and enzyme concentration was 100 nM. Error bars show standard deviations from three independent replicates.





**Figure S4.** Internal energies of the cofactor calculated during the molecular dynamic simulations. The internal bond (green), angle (orange), and dihedral (blue) energies are shown over 400 ns trajectories with (A) F_420_-2 bound to MSMEG_2027, (B) F_420_-6 bound to MSMEG_2027, (C) F_420_-2 bound to MSMEG_3380, and (D) F_420_-6 bound to MSMEG_3380.


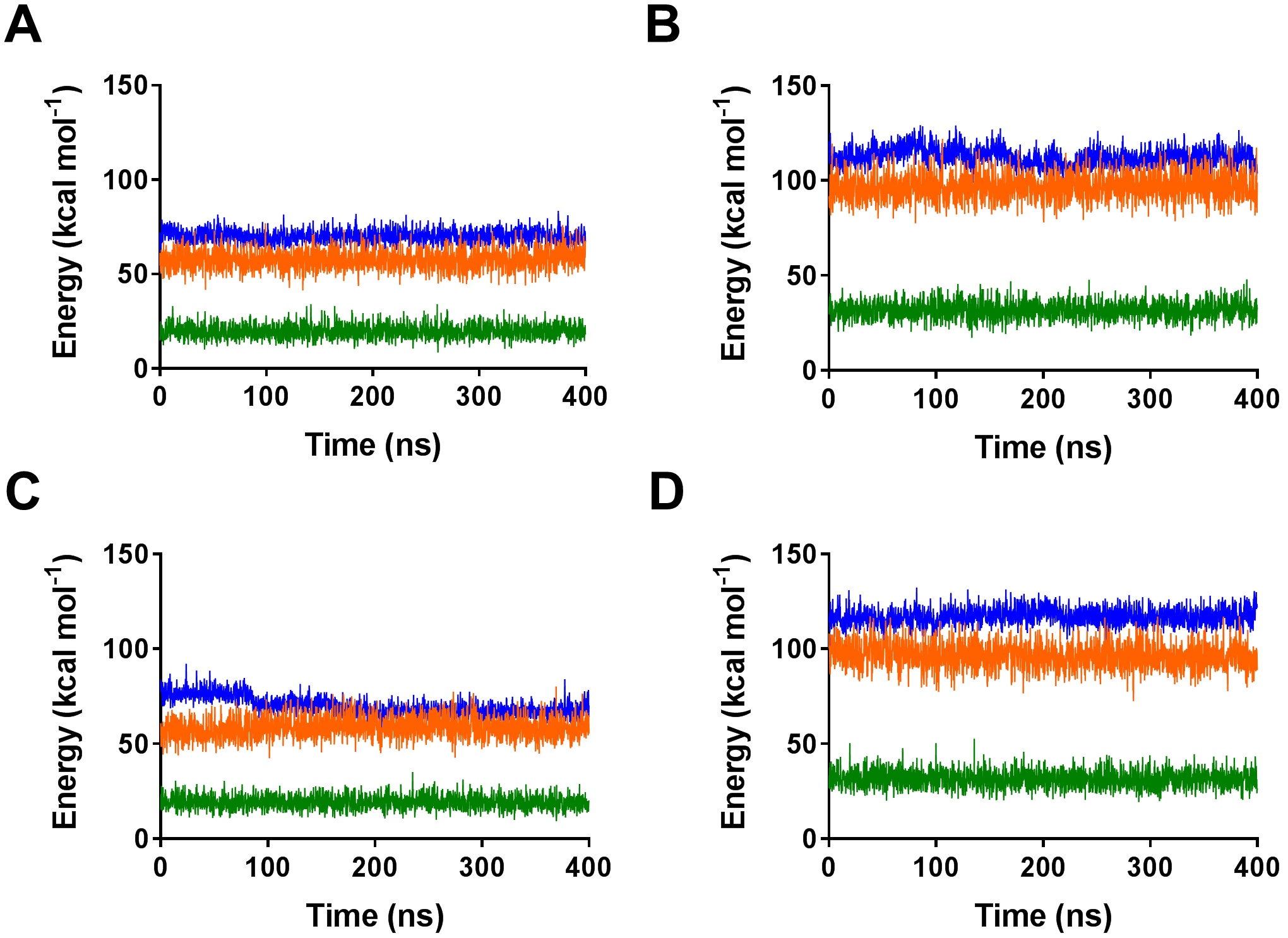


**Figure S5.** Structural basis of F_420_ oligoglutamate chain binding in F_420_-dependent oxidoreductase superfamilies. (A) Potential binding in luciferase-like hydride transferase superfamily as shown by structure of cofactor-bound Rv0407 (brown, 3B4Y) and RaptorX-constructed homology models of MSMEG_0777 (blue) and Rv0132c (minus tat signal peptide, purple). (B) Binding in flavin/deazaflavin oxidoreductase superfamily A as shown by structures of cofactor-bound Rv3548 (brown, 3R5R), MSMEG_2027 (with missing residues modeled, blue, 4Y91), and MSMEG_3356 (purple, 3H96). (C) Binding in flavin/deazaflavin oxidoreductase superfamily B as shown by structures of cofactor-bound Rv1155 (brown, 4QVB), MSMEG_3380 (blue, 3F7E), and Rv2074 (purple, 5JAB). The ribbon diagrams on the left panels also highlight the residues from MSMEG_0777, MSMEG_2027, and MSMEG_3356 implicated in binding the oligoglutamate chain. The surface renderings on the right show the hydrophobicity of the regions that bind the oligoglutamate chain. For Rv0407, due to paucity of structural information, the structure of F_420_-2 is shown and residues involved in interacting with the other four glutamate moieties are predicted.

**
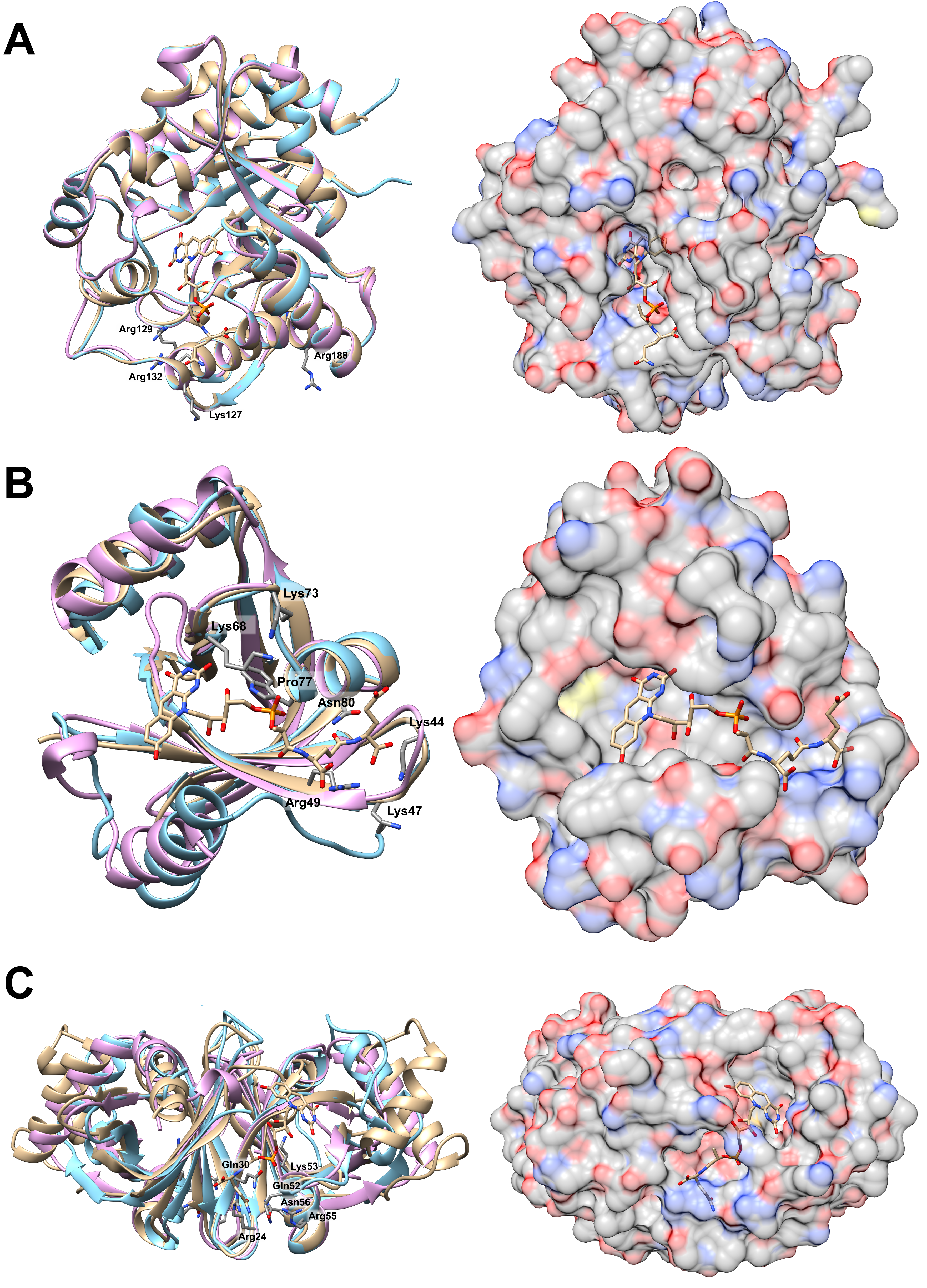
**

**Figure S6.** Multiple sequence alignments of F_420_-dependent oxidoreductase protein sequences. Sequences are shown from the luciferase-like hydride transferase superfamily (where Fgd = F_420_-dependent glucose 6-phosphate dehydrogenase, HMAD = F_420_-dependent hydroxymycolic acid dehydrogenase, HTI = F_420_H_2_-dependent dinitrophenol reductase), flavin/deazaflavin oxidoreductase superfamily A (A1, A2, A3 subgroups), and flavin/deazaflavin oxidoreductase superfamily B (B1, B2, B3, B4 subgroups). Sequences are shown from *Mycobacterium tuberculosis* H37Rv (Rv locus), *Mycobacterium smegmatis* mc^2^155 (MS locus), and *Rhodococcus opacus* PD630 (OP locus) and M213 (RS locus). Conserved or partially conserved residues implicated binding the F_420_ oligoglutamate chain are highlighted in yellow. The position of these residues is structurally mapped in **Figure S5**.

**Luciferase-like hydride transferase (LLHT) superfamily:**

MS0777 (Fgd) -------------------------------------MVAELKLGY--KASAEQFAPREL 21

Rv0407 (Fgd) --------------------------------------MAELKLGY--KASAEQFAPREL 20

OD0762 (Fgd) -------------------------------------MAHELKLGY--KASAEQFGPREL 21

MS1796 ------------------------------MHGYPQP---MTRFGY--TLMTEQSGPKDL 25

MS2456 -------------------------------------MVLSKGVGL-----FPTEPVGAM 18

Rv0132 (HMAD) MTGISRRTFGLAAGFGAIGAGGLGGGCSTRSGPTPTPEPASRGVGV--VLSHEQFRTDRL 58

RS15870 (HTI) -----------------------------------------MKVGIRIPGAGPWAGPEAI 19

.* :

MS0777 (Fgd) VELAVLAESAGMDSATVSDHFQPWRHEGGHAP------FSL----------AWMTAVGER 65

Rv0407 (Fgd) VELAVAAEAHGMDSATVSDHFQPWRHQGGHAP------FSL----------SWMTAVGER 64

OD0762 (Fgd) VELGVLAEAHGMDSATVSDHFQPWRHEGGHAP------FSL----------AWMTAVGER 65

MS1796 VHYAVAAEQAGFDFEVCSDHFSPWLTSQGHAP------NAW----------AVLGAVAHA 69

MS2456 REYVGLCESLGYDNVWFGDSQNIWRESS-----------------------TVMGAAAVG 55

Rv0132 (HMAD) VAHAQAAEQAGFRYVWASDHLQPWQDNEGHSM------FPW----------LTLALVGNS 102

RS15870 (HTI) TEVSRFAEKIGFDSLWMTDHVALPTRVETAYPYTDDGKFLWDPATPYLDCLTSLTWAAAA 79

.* * * : ..

MS0777 (Fgd) TKNLVLGTSVLTPTFRYNPAV-IAQAFATMGCLYPGRIFLGVGTGEALNEIATGYAGEWP 124

Rv0407 (Fgd) TNRLLLGTSVLTPTFRYNPAV-IAQAFATMGCLYPNRVFLGVGTGEALNEIATGYEGAWP 123

OD0762 (Fgd) TERLQLGTSVMTPTFRYNPAV-VAQAFATMGCLYPGRIMLGVGTGEALNEIATGFAGEWP 124

MS1796 TESAHLYSYVTCPTMRYHPAV-VAQQAATVQILSDGRFTLGLGSGENLNEHVV--GKGWP 126

MS2456 TERIVFGTGVTNAVTRHTSL--LASTWATLAEFTGGRVALGIGTGDSSLRT-------MG 106

Rv0132 (HMAD) TSSILFGTGVTCPIYRYHPAT-VAQAFASLAILNPGRVFLGLGTGERLNEQAA--TDTFG 159

RS15870 (HTI) TERMELGTS--CLILPWRPLVQTAKTLVSIDVMSRGRLSVAIGVGWMKEQF----ELLGA 133

*. : : *. .:: : .*. :.:* * .

MS0777 (Fgd) EFKERFARLRESVRLMRELWLGDRVDFDG--EYYRTKGASIYDVPE-GGIPVYIAAGGPV 181

Rv0407 (Fgd) EFKERFARLRESVGLMRQLWSGDRVDFDG--DYYRLKGASIYDVPD-GGVPVYIAAGGPA 180

OD0762 (Fgd) EFKERFARLREAVRLMRELWLGDRVDFEG--EYFTTKGASIYDVPE-GGIPVYIAAGGPV 181

MS1796 TVERRLDMLAEAIKIIRELFTGELIDFRG--EYFEVDSARIWDVPD-EPVGIGVSLTGER 183

MS2456 LKPQRLAELEKSVSDLRALFRGEKVAEATSGAEYHL-----NYLSEPVDIPIYIAASAPK 161

Rv0132 (HMAD) NYRERHDRLIEAIVLIRQLWSGERISFTG--HYFRTDELKLYDTPA-MPPPIFVAASGPQ 216

RS15870 (HTI) PFKDRGKRTTEMVNAMRHMWKEDEVAFDG--EFYQLHDFKMYPKPVRGTIPVWFAGYSTA 191

* : : :* :: : : : : .: .

MS0777 (Fgd) VAKYAGRAGDGFICTSGKGEELYAEKLIPAVKEGAAAADRDADAI----DRMIEIKISYD 237

Rv0407 (Fgd) VAKYAGRAGDGFICTSGKGEELYTEKLMPAVREGAAAADRSVDGI----DKMIEIKISYD 236

OD0762 (Fgd) VARYAGRSGDGFICTSGKGMELYTDKLMPAVAEGAEKADRDVAEI----DKMIEIKISYD 237

MS1796 ALEKLAVSTDHLINVAPD----------AAVVEGWRK-RREATGILPEGRVVGQIPVCWD 232

MS2456 ILRMSGRIADGVIVLVGTAPH-FIEAALETIAAGAAESGRTLDDLH----IVLWTPTAID 216

Rv0132 (HMAD) SATLAGRYGDGWIAQAR---DINDAKLLAAFAAGAQAAGRDPTTL----GKRAELFAVVG 269

RS15870 (HTI) SLRRIAAIGDGWHPLAIGPEE-YAG-YLATLKQYAEEAGRDMNEITLTARPLRKAP--YN 247

. * :. * : .

MS0777 (Fgd) TDPELAL---ENTRF---WAPLSLTA--EQKHSIDDPI----------EMEKAADALPIE 279

Rv0407 (Fgd) PDPELAL---NNTRF---WAPLSLTA--EQKHSIDDPI----------EMEKAADALPIE 278

OD0762 (Fgd) TDPELAL---ENTRF---WAPLSLTP--EQKHSIDDPI----------EMEKAADALPIE 279

MS1796 PDKDAAIA--RAHDQ---FRWFAGGW--AVNADLPTPA----------GFAAATQFVRPE 275

MS2456 DDRTKARDLVRAHVSRVAIRPLPAKVAPELEEAIDRIRKSYNYYEHMNTEASHADLVPDE 276

Rv0132 (HMAD) DD-KAAA---RAADL---WRFTAG------AVDQPNPV----------EIQRAAESNPIE 306

RS15870 (HTI) AETIEAYGELGVTHF---ICDTSFEH-DTLEATMDELA----------ELADA--VLPTA 291

: *

MS0777 (Fgd) QVAKRWIVASDPDEAVEKVGQYVKWGLNHLVFHA-PGHDQRRFLELFKRDLEPRLRKLA- 337

Rv0407 (Fgd) QIAKRWIVASDPDEAVEKVGQYVTWGLNHLVFHA-PGHDQRRFLELFQSDLAPRLRRLG- 336

OD0762 (Fgd) QVAKRWIVASDPDEAVAQIRPYLDAGLNHLVFHA-PGHDQKRFLELFERDLAPRLRG--- 335

MS1796 DVASAIPCGPDLDAIVAAVDEYRQAGFTDIALIQIGGDSQDQFLAEAATPLLAALRESAG 335

MS2456 -LVDLFALAGTPAECGQRLKEIEALGVDQVSIVPFVRPGESRAPTIRTFADLV-----GG 332

Rv0132 (HMAD) KVLANWAVGTDPGVHIGAVQAVLDAGAVP-FLHF-PQDDPITAIDFYRTNVLPELR--HG 360

RS15870 (HTI) ---HNLP----------------------------------------------------- 295

**Flavin/deazaflavin oxidoreductase A (FDOR-A) superfamily:**

MS2027 (A1) ----------------MTDAELSPTDWVRE-----------------QTERI-LE--QGT 24

Rv3547 (A1) -------MPKSPPRFLNSPLSDFFIKWMSR-----------------INTWM-YRRNDGE 35

MS2850 (A1) --------------------MTEAIDWDQM-----------------NNQVIKEFRETGG 23

MS5998 (A1) MADTSRPLNAKQLERLNAKSTGTLIKWMSR-----------------FQTFL-FKTTNGK 42

OP5945 (A1) -------------MPLTGEYEPSPSKWAAD-----------------QAELM-ES--TDG 27

Rv1261c(A2) ---------------------MDISRWLERHVG---------VQLLRLHDAI-YRGTNGR 29

MS5030 (A2) ------------------------MPWWERYIG---------LPLLLLHDKV-YKATDGR 26

MS6325 (A3) MDDKLHGIPRVDLETRP-RWKRDLAWWFGGKVLATARASAIWRKIAMPYEVPLIKATGGR 59

MS5215 (A3) -----MPLPYVDPHKKR-GLGYEIASAFGRSPVGQ----FFARHVARHTDPLLFRLTRGR 50

OP6325 (A3) -------------------------------MF---------VKALQLHQFV-YERSGGW 19

.

MS2027 (A1) TDGVHVLDRPIVLFTTTGAKSGKKRYVPLMRVEENGKYAMVASKGGDPKHPSWYFNVKAN 84

Rv3547 (A1) GLGGTFQKIPVALLTTTGRKTGQPRVNPLYFLRDGGRVIVAASKGGAEKNPMWYLNLKAN 95

MS2850 (A1) KAGGLFEGSPLVLVHHTGAKSGKQRIAPLVPLLDGDRIYIFGSKGGADSHPDWYHNLVAN 83

MS5998 (A1) LGNKFLRGTEVGILTTIGRKSGEPRDTPLLFLQEGRRIVLVASQGGRATNPMWYLNLKAN 102

OP5945 (A1) AKGTTLGGRPVVLLTTRGAKSGKLRKTPLMRVEHDGTYAIVASLGGAPQNPVWYYNVKAE 87

Rv1261c(A2) IGHRIPGAPPSLLLHTTGAKTSQPRTTSLTYARDGDAYLIVASKGGDPRSPGWYHNLKAN 89

MS5030 (A2) IGHRIPGGPATLILHTVGAKTGQHRASSLAYARDGDDYLVVASKGGEPKAPGWYHNLKAD 86

MS6325 (A3) A--RLSVGIPIAVLTSTGARSGKTRQTALAYFTDGDDVVLIASNYGQARHPGWYHNLRAH 117

MS5215 (A3) I--NVSP-IINAPLVTTGAKSGERREVQLTYFHDHDDVILVASNFGGTKNPQWYHNLKAH 107

OP6325 (A3) VGHRLLFGMPTLLLHNVGRKSGQPRTSALTYGRDGARFLVVASKGGAPTSPAWMHNVLAA 79

. * ::.: * * . : .* * * * *: *

MS2027 (A1) PTVSVQDGDKVLPDRTARELEGE--EREHWWKLAVEA---YPPYAEYQTK---TDRLIPV 136

Rv3547 (A1) PKVQVQIKKEV-LDLTARDATDE--ERAEYWPQLVTM---YPSYQDYQSW---TDRTIPI 146

MS2850 (A1) PDTVVELGTET-FPVKARVLTGA--ERDEIYAKQVAV---APQFGDYQRK---TTRVIPV 134

MS5998 (A1) PKVTFQTRSEK-LALVAREATDA--ERDEYWPKLDAM---YPDFANYRSY---TDRKIPI 153

OP5945 (A1) PHVELRDGSTT-QDMVAREVTGE--EKTAWWDRAVAA---FPDYAEYQKK---TDREIPV 138

Rv1261c(A2) PDVEINVGPKR-FGVTAKPVQPHDPDYARLWQIVNEN--NANRYTNYQSR---TSRPIPV 143

MS5030 (A2) PNVEINVGPKR-LRATARAVFPDDPDFPRLWEIVNNMPGNKDRYIGYQKR---TTRQIPV 142

MS6325 (A3) PECELYVGRRG-GRFVAREVDGP--QRDRLYALAASR--LYPGWVAYEKR-AEGVRRIPV 171

MS5215 (A3) PDCEFG----G-EPFTAAEVTDP--DEHARLYALAER--VYPGYRDYREATADTGRHIPV 158

OP6325 (A3) PTCEIQVGRDH-ITVAARKVLPGDPDYERMWATMNAI--NHGRYSDYQKK---TDRPIPV 133

* . * : : *. * **:

MS2027 (A1) FIVE---- 140

Rv3547 (A1) VVCEP--- 151

MS2850 (A1) VELQRV-- 140

MS5998 (A1) VICDPA-- 159

OP5945 (A1) FVLEPA-- 144

Rv1261c(A2) VVLTRR-- 149

MS5030 (A2) IVLTPVS- 149

MS6325 (A3) LRLTPADP 179

MS5215 (A3) FRLTPR-- 164

OP6325 (A3) IALTPTR- 140

.

**Flavin/deazaflavin oxidoreductase B (FDOR-B) superfamily:**

MS3380 (B1) --------MVAVPEGYESLLERPLYGHLATVRPDGTPQVNAMWFAWDGE-------VLRF 45

MS0048 (B1) MGKNERTKIVMSDEEIAEFVERSRTATMATVLPDGRPHLVAMWYAVVDG-------EIWF 53

Rv2991 (B1) MGTKQRADIVMSEAEIADFVNSSRTGTLATIGPDGQPHLTAMWYAVIDG-------EIWL 53

OP3947 (B1) MGKNQRSQITMTDSEIAEFVDRSRIATLATVAADGRPHLVAMWYAVIDG-------EIWF 53

MS6526 (B2) ----------MAEFDAVTAFADAPAAVLSTLNADGAPHLVPVVFAVHVPHVEGQPARIYT 50

MS5170 (B3) ------MGRQVFDDKLLALICNNSLGVLATIKQDGRPQLSNVSYHFDPRA-----QTFQV 49

Rv1155 (B3) ------MARQVFDDKLLAVISGNSIGVLATIKHDGRPQLSNVQYHFDPRK-----LLIQV 49

MS3880 (B4) MAASRGKATTRLTTDALAFLTERHLAMLTTLRSDGSPHVVAVGFTFDPKT-----HIARV 55

Rv2074 (B4) --MAMVNTTTRLSDDALAFLSERHLAMLTTLRADNSPHVVAVGFTFDPKT-----HIARV 53

. . ::*: *. *:: : :

MS3380 (B1) THTTKRQK------YRNIKANPAVAMSVIDPDNPYRYLEVRGL----VEDIVPDPTGA-- 93

MS0048 (B1) ETKAKSQK------AVNLRRDPTVTVLIEDGH-TY--DTLRGVSIDGTAEIVDDPETL-- 102

Rv2991 (B1) ETKAKSQK------AVNLRRDPRVSFLLEDGD-TY--DTLRGVSFEGVAEIVEEPEAL-- 102

OP3947 (B1) ETKSKSQK------AVNLRRDDRVTVLIEDGQ-TY--DTLRGISIEGRAEIVEDPDAM-- 102

MS6526 (B2) AVDAKRKTTRNLRRLANIDRDSRVSLLVDHYSDDW--TQLWWVRADGVATTHHSGDEVAT 108

MS5170 (B3) SITEPRAK------TRNLRRDPRASIHVSSDD-GWAYAVAEGDAILTPPAASTHDDTVEG 102

Rv1155 (B3) SIAEPRAK------TRNLRRDPRASILVDADD-GWSYAVAEGTAQLTPPAAAPDDDTVEA 102

MS3880 (B4) ITTGGSQK------AVNAQERGVAVLSQVDGA--------RWLSLEGKSTVSSDPDAV-- 99

Rv2074 (B4) ITTGGSQK------AVNADRSGLAVLSQVDGA--------RWLSLEGRAAVNSDIDAV-- 97

. * . .

MS3380 (B1) ---------------------FY--LKLNDRYDGPLTEPPADKADRVII---VVR--P-- 123

MS0048 (B1) ---------------------LRVGISVWERYTGPYTDEMRPFVDQMMNNRIAVRVVPGR 141

Rv2991 (B1) ---------------------HRVGVSVWERYTGPYTDECKPMVDQMMNKRVGVRIVARR 141

OP3947 (B1) ---------------------FAVGVSVWERYTGPYTDDLRPAIDQLLYKRVVVRIVPER 141

MS6526 (B2) GYALLRAKYHQYERVSLDGPVISVEVSRWASW---------------------------- 140

MS5170 (B3) LIALYRN--------------ISGEHPDWDEFRQAMVDDRRVLMTLPITHVYGMPPGM-- 146

Rv1155 (B3) LIALYRN--------------IAGEHSDWDDYRQAMVTDRRVLLTLPISHVYGLPPGM-- 146

MS3880 (B4) ---------------------RDAELRYAQRYRTPRVNPRRVVIEVRIERVLGSSELLDR 138

Rv2074 (B4) ---------------------RDAELRYAQRYRTPRPNPRRVVIEVQIERVLGSADLLDR 136

:

MS3380 (B1) -TAFSKQ------------------------ 129

MS0048 (B1) TRSWDHRKLGMPAMPLGGSTAQYLNS----- 167

Rv2991 (B1) TRSWDHRKLGLPHMSVGGSTAP--------- 163

OP3947 (B1) IRSWDHAKLGMPAMPVGGTTAAYLETADRRA 172

MS6526 (B2) ------------------------------- 140

MS5170 (B3) -R----------------------------- 147

Rv1155 (B3) -R----------------------------- 147

MS3880 (B4) S------------------------------ 139

Rv2074 (B4) A------------------------------ 137
